# Evaluation of the mechanical behavior of concrete reinforced with waste tire steel fibers

**DOI:** 10.1038/s41598-025-07615-0

**Published:** 2025-10-10

**Authors:** Ahmed Faisal Oan, Fatma Attia

**Affiliations:** 1 Construction Engineering Department, Faculty of Engineering, Egyptian-Russian University, Cairo, Egypt; 2 Civil Engineering Programme, School of Engineering and Computer Science, University of Hertfordshire Hosted by GAF, Cairo, Egypt; 3 Department of Structural Engineering, Faculty of Engineering, Cairo University (CU), Giza, Egypt

**Keywords:** Engineering, Civil engineering

## Abstract

Concrete serves as the foundation of modern infrastructure and is commonly used in construction for its strength, durability, and versatility. However, traditional concrete has its limitations, especially when it comes to handling tensile stresses. To address this issue, different types of fiber reinforcement have been developed, with steel fiber proving to be particularly effective due to its high tensile strength and compatibility with concrete. This study investigated the effect of using waste tire steel fiber (WTSF) on the mechanical properties of concrete, where different percentages of WTSF were used namely, 0.25%, 0.5% and 1% by volume replacement of concrete. Compared to the control mix, the incorporation of steel fibers, whether commercially manufactured (MSF) or recycled (WTSF), negatively affected workability. Slump values in MSF mixes decreased between 37.5% and 62.5%, while WTSF mixes showed higher reductions, ranging from 87.5 to 100%, indicating that WTSF had a more adverse impact on workability. The compressive strength results showed that using WTSF at volume ratios of 0.25%, 0.5%, and 1% led to reductions of 6.4%, 30%, and 46%, respectively. In contrast, incorporating steel fiber enhanced mechanical performance, with tensile strength increasing by up to 67% for MSF and 38% for WTSF, while flexural strength improved by up to 40% and 19%, respectively.

## Introduction

Steel fibers reinforced concrete (SFRC) is one of the commonly utilized structural materials. For decades, manufactured steel fibers (MSF) have been added to concrete mixes as a better alternative to traditional reinforcement rebar mesh. Unlike mesh reinforcement, MSF can be uniformly distributed within structural elements. This distribution provides isotropic reinforcement and eliminates potential weak planes where cracks may develop. Research has shown that incorporating MSF into concrete enhances ductility and helps control crack propagation^1,2^. Although the rates of improvement may vary, SFRC demonstrates superior post-cracking flexural strength, enhanced fracture resistance, higher fatigue resistance, increased resistance to spalling, and greater first-crack strength^3–7^ .

Statistics showed that there is a significant increase in demand for MSF worldwide, where about 300,000 tons per year have been produced to satisfy the needs of the construction industry. Moreover, this number is expected to rise by 20% each year^8^. Steel fiber manufacturing is energy-intensive. Many steel companies worldwide produce large quantities of MSF, which consume fossil fuels and emit considerable amounts of carbon dioxide. Due to the environmental concerns and depletion of natural resources associated with large-scale MSF production, extensive research has been devoted to finding sustainable alternatives. Studies have suggested that using recycled steel fibers is a promising solution, as it reduces environmental impact and lowers recycling costs^9–11^.

Annually, over one billion tires reach the end of life around the world^12^. Over 50% of the waste tires (WT) are disposed of without undergoing any treatment, primarily by combustion or dumping^13^. Effectively disposing of large quantities of WT is a significant difficulty. Due to their significant environmental impact, the disposal of these tires through landfilling is not allowed in Europe and the US^8,12^. WT has the potential to be recycled and utilized as fuel in cement furnaces, as construction material in asphalt pavements or employed as recycled aggregates in concrete mixes^14,15^. The steel fibers in WT can be extracted using shredding, freezing, or thermal decomposition procedures during the recycling of worn tires^9^. Over half a million tons of steel fibersmight be yearly obtained in Europe through the recycling of worn tires^12,16^.

The significant environmental and safety concerns associated with the large volume of waste tires (WT) have encouraged researchers to explore the use of steel fibers extracted from WT as a sustainable alternative to manufactured steel fibers (MSF). WTSF is considered an economical option, as it is typically a low-value by-product with limited market applications and is often discarded. In contrast, MSF involves high production costs and contributes to environmental degradation due to substantial carbon emissions generated during large-scale manufacturing. Therefore, incorporating WTSF into concrete not only reduces waste but also offers economic and environmental benefits by minimizing the demand for MSF.

 Samarakoon et al.^17^, performed experimental studies to investigate the fresh and hardened properties (SFRC) produced by reutilizing waste tire steel fibers (WTSF) as a sustainable alternative to (MSF). Samples with WTSF and MSF ratios of 0.5% and 1% by volume were evaluated and compared with plain concrete samples. The results indicated that increasing the fibers content has led to a decrease in the slump values of the SFRC. The compressive strength of the MSF showed an increase between 17 and 20% while only increased by 5 to 12% for the samples of WTSF. They reported that increasing the WTSF content by 0.5% and 1% can enhance concrete behaviour in the post-cracking. However, the MSF showed a more significant impact on the post-cracking behaviour. Moreover, a four-point beam test has been conducted, and the results demonstrate that adding 0.5% WTSF has improved the ductility while the MSF showed better results for the same steel fibers ratio. Furthermore, both beam samples of 1% WTSF and 1% MSF showed a high level of concurrence, recording a mid-span deflection of 91.7 mm and 97.7 mm, respectively.

Zia et al.^18^, performed experimental work to assess the properties of SFRC samples made using WTSF. The investigated ratios of raw WTSF were varied between 0.30%, to 0.75% by the concrete volume. The results showed a reduction of 37%, 64%, and 67% in slump test results for the samples of 0.30%, 0.5% and 0.75% WTSF ratio, respectively compared to the plain concrete sample. The addition of 0.3% WTSF by volume of concrete resulted in a 20% improvement in the compressive strength. However, there was an observed drop of 8% in compressive strength for the 0.45% WTSF ratio and a decrease of 17% for the 0.75% WTSF ratio. Moreover, the addition of 0.3% WTSF by volume to concrete resulted in a maximum improvement of 16% in splitting tensile strength. When comparing, it was observed that there is a 5% increase for the 0.5% WTSF ratio and a 4% increase for the 0.7% WTSF ratio. The study recommended that further experimental work is required to investigate the impact of using ratios higher than 0.75% WTSF on concrete properties.

Su et al.^19^ performed an experimental investigation to assess the mechanical characteristics and durability of SFRC with WTSF as a sustainable alternative to MSF. Samples of MSF were examined with an addition of 0.5% by volume, whereas WTSF was incorporated at ratios of 0.5%, 1.0%, and 1.5%. The results indicated that, at the same steel fiber ratio, the WTSF sample exhibited enhanced workability relative to the MSF samples. MSF, with a ratio of 0.5%, exhibited a significant reduction in slump test results of 83.5%, whereas WTSF at the same ratio demonstrated only a 9.0% reduction compared to plain concrete samples. Increasing the WTSF ratio by 1.0% and 1.5% has resulted in a slump reduction of 12.2% and 19.1%, respectively, compared to plain concrete samples. Both WTSF and MSF exhibited a marginal enhancement in concrete compressive strength, ranging from 2.4 to 4.0% at 28 days, in comparison to control samples. Results of the splitting tensile strength test for the WTSF and MSF exhibit closely aligned results, demonstrating enhancements of 15.4% and 16.9%, respectively, in comparison to plain concrete. The WTSF exhibited superior durability performance relative to MSF and plain concrete samples. Samples containing WTSF and MSF at a replacement ratio of 0.5% exhibited a decrease of 8.3% and 6.3% in drying shrinkage at 14 days, respectively, compared to plain concrete.

Dŏgruyol et al.^3^ Performed an experimental investigation on the properties of WTSF concrete subjected to elevated temperatures. The WTSF was added to concrete at 0.4% and 0.8% by volume as a replacement for fine aggregate, while the samples were tested at room temperature (25ºC) and at 400, 600, and 800ºC to simulate fire conditions. The findings indicated that increasing percentage of WTSF decreased both homogeneity and workability. The compressive strength of the WTSF concrete at 25ºC decreased by 1.72% and 6.57% for samples with 0.4% and 0.8% WTSF ratios, respectively, in comparison to the plain concrete sample. At 800 ºC, the compressive strength has increased by 31.65% and 16.69% for WTSF ratios of 0.4% and 0.8%, respectively.

Several studies have investigated the mechanical behaviour of concrete reinforced with waste tire steel fibers (WTSF); however, the findings remain inconsistent, particularly with respect to compressive strength. Hu et al.^20^ and Su et al.^19^ observed insignificant improvements in compressive strength. Su et al.^19^ reported that both WTSF and manufactured steel fibers (MSF) resulted in slight improvements in compressive strength, ranging from 2.4 to 4%, suggesting a negligible effect. In contrast, other researchers, such as Papakonstantinou et al.^20^ and Leone et al.^21^, observed a reduction in compressive strength associated with WTSF incorporation. These discrepancies may be attributed to differences in fiber geometry, aspect ratio, content, and mixing methods, as well as the presence of residual rubber on the fiber surface. Moreover, Papakonstantinou et al.^21^ and Leone et al.^22^ and Dŏgruyol et al.^3^ indicated that the compressive strength decreased further when WTSF were added. Regarding workability, Papakonstantinou et al.^21^ observed that SFRC made of WTSF exhibited enhanced workability when the fibre content was below 4%. Observations by Papakonstantinou et al.^21^ and Bjegovicet al.^23^ indicate that the modulus of elasticity does not considerably increase with higher replacement ratios of steel fiber reinforcement. Leone et al.^22^ identified that the splitting tensile strength of steel fiber reinforced concrete made of both WTSF and MSF has marginally reduced in comparison to the control concrete sample. Conversely, Su et al.^19^ reported that WTSF and MSF exhibit closely aligned results, demonstrating enhancements of splitting tensile strength.

Despite the growing interest in using waste tire steel fibers (WTSF) as a sustainable alternative to manufactured steel fibers (MSF), there remains a lack of consensus regarding its impact on the mechanical properties of concrete. This highlights a critical research gap. It is essential to establish whether concrete reinforced with WTSF performs comparably to that reinforced with MSF and remains within acceptable performance ranges. Limited studies have explored the mechanical behaviour of concrete containing higher ratios of WTSF, particularly in the post-cracking phase. Therefore, this study aims to systematically assess the mechanical performance of concrete incorporating varying ratios of WTSF and compare it with MSF-reinforced concrete. Special emphasis is placed on evaluating workability, compressive strength, tensile strength, and flexural strength to address and clarify the conflicting results reported in existing literature.

## Experimental program

The experimental program involved testing a total of 84 specimens, which were divided into three categories. The first category consisted of standard cubes 150 × 150 × 150 mm, used for compression tests at both 7-day and 28-day ages. The second category included small cylinders with a height of 200 mm and a diameter of 100 mm, used for indirect split tension tests. The third category comprised beams with a rectangular cross-section of 150 × 150 mm and a length of 750 mm, used for flexural tests. Splitting tensile and flexural tests were conducted at the 28-day age. For all tests carried out in the experimental program, three replicates from each specimen were tested and the average values are used for the analysis.

The specimens were categorized into three different categories; the first category represented the control specimens with no added steel fibers (plain concrete), the second category was the concrete mixturereinforced with waste tire steel fiber (WTSF), while the last category was the concrete mixture reinforced by manufactured steel fiber (MSF). The WTSF and MSF were added as percentages of concrete volume, where the steel fibers percentages used in this study were 0.25%, 0.5%, and 1.0% by volume of concrete.

### Mix design

The concrete mix was designed using the BRE method, aiming for a target compressive strength of 30 MPa at 28 days. The concrete mix proportions were determined according to the guidelines outlined in ECP 203^24^. Steel fibers were incorporated into the mix at volumes of 0.25%, 0.5%, and 1.0%, by volume of concrete, for both WTSF and MSF. Table 1 shows the mix proportions for different concrete mixtures.

**Table 1:** Mix proportions.

Mix	Cement (kg/m^3^)	Fine Aggregates (kg/m^3^)	Coarse Aggregate (kg/m^3^)	Water (Lit/m^3^)	WTSF (kg/m^3^)	MSF (kg/m^3^)
Control	405	681	1065	225	-	-
WTSF 0.25%	405	681	1065	225	5.93	-
WTSF 0.50%	405	681	1065	225	11.87	-
WTSF 1%	405	681	1065	225	23.75	-
MSF 0.25%	405	681	1065	225	-	5.93
MSF 0.5%	405	681	1065	225	-	11.87
MSF 1%	405	681	1065	225	-	23.75

### Materials

The cement used in this study was CEM I 42.5 N Pozzolanic from Sinai Company, Egypt. The coarse aggregate used was dolomite crushed stone with a maximum size of 20 mm, and siliceous sand was used for fine aggregates. Potable water was used for mixing the concrete. Sieve analysis test was carried out according to (ESS) No. 1109-1/2002 to ensure that the aggregate grading is within the acceptable limit.

**Fig. 1:**
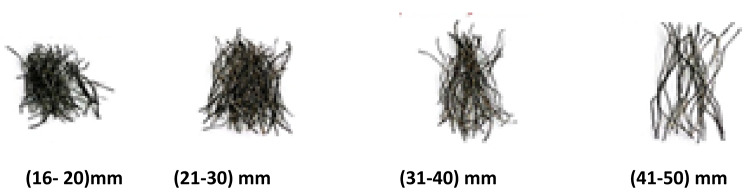
WTSF extracted from the tire recycling process.

**Fig. 2:**
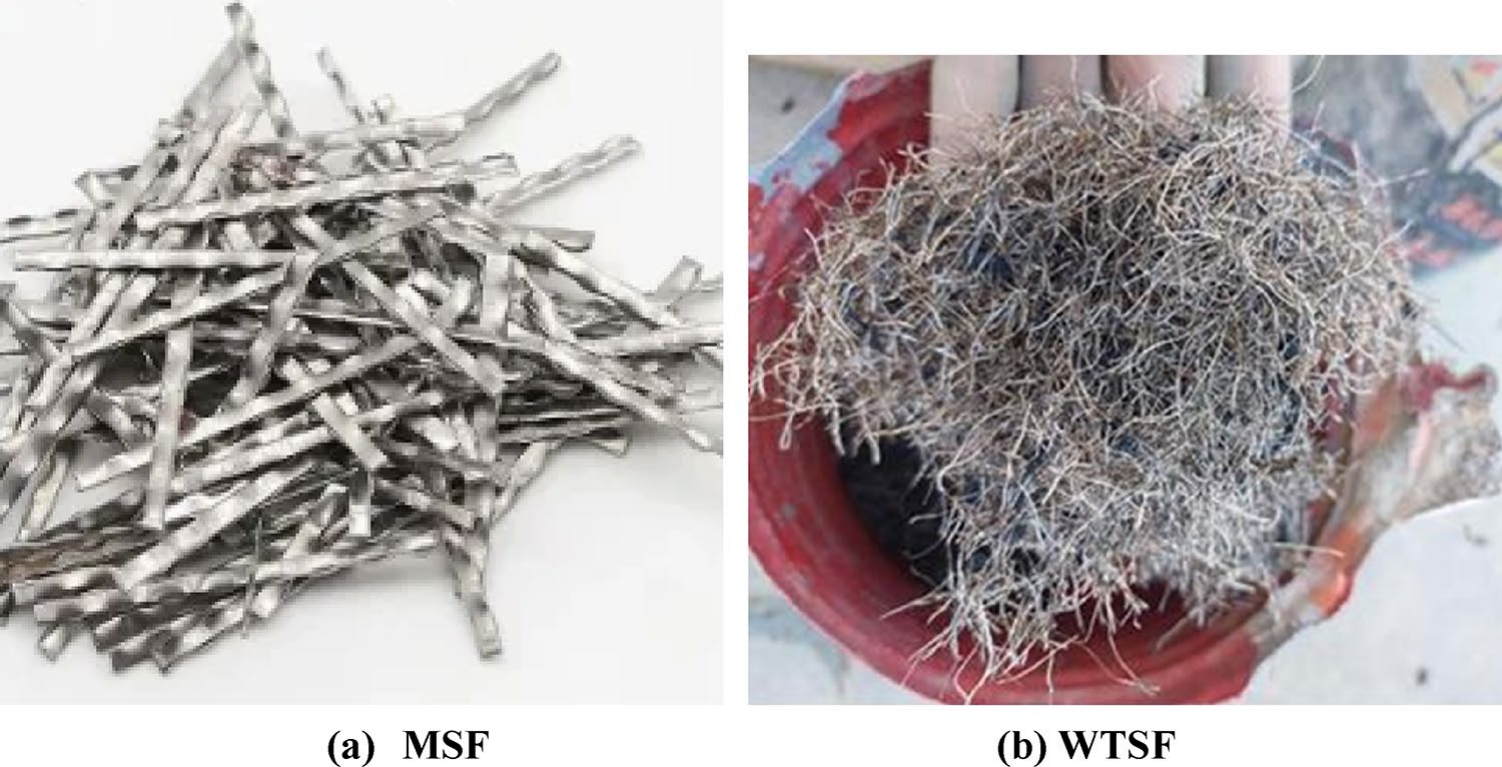
Types of steel fibers used.

WTSF were sourced from a national tire recycling facility. The recycling process involves cutting tires into sections or shredding them into smaller chips, typically 16–50 mm in length and about 0.3 mm in diameter resulting in a range of aspect ratios between 50 and 170. The steel fibers are then extracted from the shredded material using magnetic separation method, as the steel is magnetic and can be easily separated from non-magnetic components such as rubber and textiles. Once extracted, the steel fibers undergo a cleaning process to remove any residual contaminants or tire remnants. This may include washing, drying, or additional treatment to ensure the fibers are free of impurities. Figure 1 shows the shapes of WTSF obtained. While the used MSF were SikaFiber^®^ Novocon^®^ XR with a length of 50 mm and a thickness of 1.14 mm. Figure 2 shows the two types of manufactured and waste steel fibers used.

In order to minimize the expecting agglomeration of WTSF in the concrete mixtures, the following techniques were adopted.

- Manual separation of WTSF : The fibers were manually loosened to break up clusters which helped the initial agglomeration tendency.
- Mixing process: A two-stage mixing process was used; first dry components (cement, sand, and coarse aggregates) were blended, followed by the addition of WSF and then water and admixtures. Prolonged mixing (within practical limits) after fiber addition helped achieve better dispersion.
- Gradual addition of fibers during mixing which prevented the formation of fiber balls.

## Results and discussion

### Workability

In this study, workability was assessed through conducting the slump test for different concrete mixtures. The slump test wascarried out on the different concrete mixtures. Table 2 shows the obtained slump test according to ASTM C143^25^ values for different concrete mixtures.

**Fig. 3:**
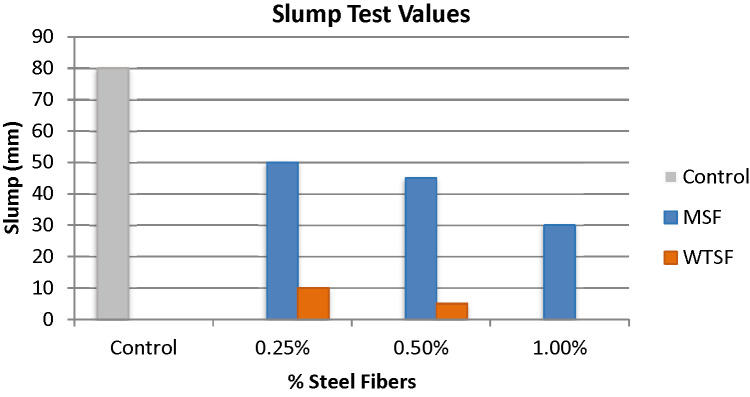
Relation between slump test values and the ratios of MSF and WTSF used in the tested concrete mixture samples.

**Fig. 4:**
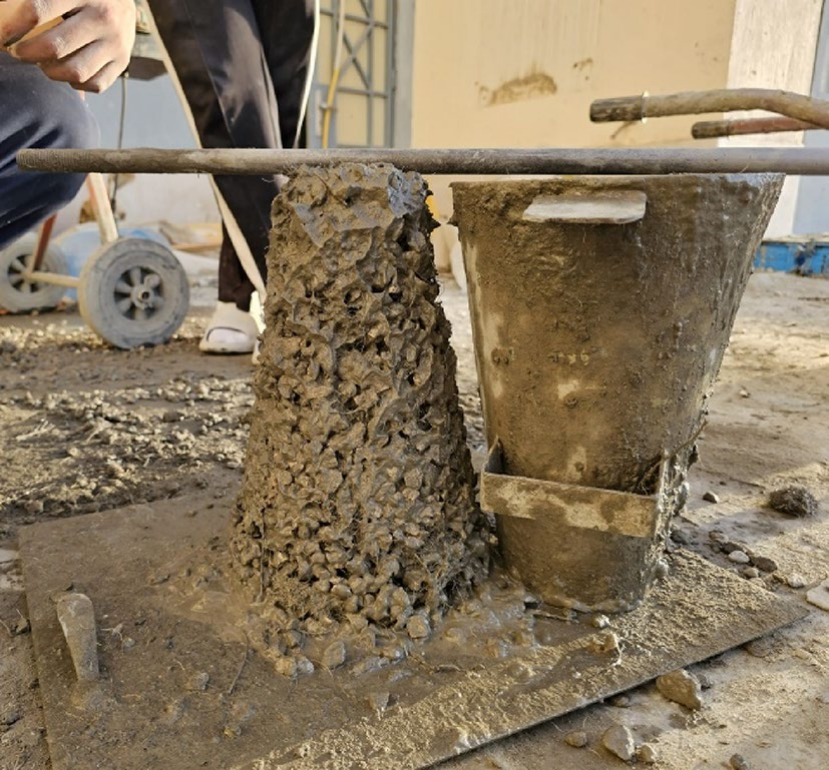
Slump test (1% WTSF).

Both types of steel fibers showed a significant reduction in slump when added to the concrete mixture, and by increasing the percentage of fibers in the concrete mixture the reduction in slump increases. The relation between slump test values and the percentage of MSF and WTSF for the tested concrete mixtures samples is illustrated in Fig. 3. The samples incorporating MSF showed a reduction in slump values of 37.5%, 43.8%, and 62.5% for the mixes with 0.25%, 0.50%, and 1% of MSF, respectively, in comparison to the control sample. However, the samples incorporating WTSF showed a reduction in slump values of 87.5%, 93.8% and 100% for the mixes with 0.25%, 0.50%, and 1% of WTSF, respectively, relative to the control sample. The degradation in slump was less when using MSF compared to that of the WTSF. The reason for that is the fact that the MSF were better distributed within the concrete mix compared to the WTSF which was likely to form clumps in the concrete and as the percentage of the steel fibers increase the tendency to form such clumps increases, and hence the slump drops drastically until it becomes zero slump at 1% WTSF. Also, WTSF often has a high aspect ratio and may be curved or twisted, creating physical interlocking which restricts the movement of fresh mix particles in the concrete mixtures. Similar results were reported by Sengul^26^, Zia et al.^18^. Figure 4 shows the slump of the concrete mix with 1% WTSF.

**Table 2:** Slump test Values.

Concrete mixture	Slump (mm)
Control	80
MSF (0.25%)	50
MSF (0.5%)	45
MSF (1%)	30
WTSF (0.25%)	10
WTSF (0.5%)	5
WTSF (1%)	0

### Modulus of elasticity

**Fig. 5:**
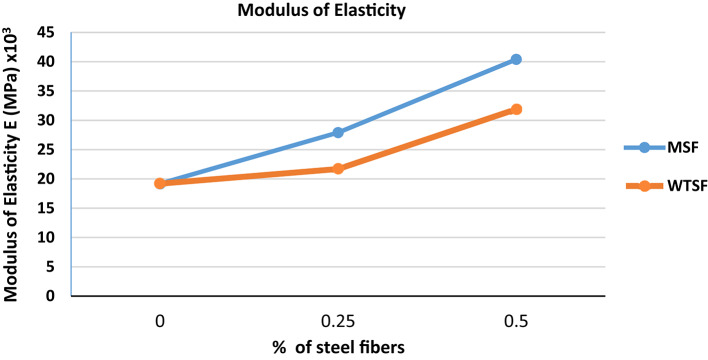
Modulus of Elasticity of different concrete mixtures.

The modulus of elasticity was measured for the control, 0.25% and 0.5% specimens according to ASTM E111^27^ investigate the effect of adding different types of steel fibers on the modulus of elasticity of concrete, the results are shown in Table 3. The results indicate a significant increase in the modulus of elasticity of concrete, compared to the control sample, upon the incorporation of steel fibers (in both forms) at a rate of 0.25%. This enhancement was noticeable as the percentage of steel fibers increased from 0.25 to 0.50%, as illustrated in Fig. 5. When comparing the findings of this study with those reported by S.M.S.M.K. Samarakoon et al.^17^ it is observed that while they noted an improvement in the modulus of elasticity with the incorporation of MSF and WTSF, the improvement rate was described as minimal and considered insignificant.

**Table 3:** Modulus of elasticity of different concrete mixtures.

Mix	Modulus of elasticity (MPa)
Control	19.2 × 10^3^
0.25% WTSF	21.7 × 10^3^
0.50% WTSF	31.9 × 10^3^
0.25%MSF	27.9 × 10^3^
0.50%MSF	40.4 × 10^3^

### Compressive strength

The compression test was carried out on standard cubes 150 × 150 × 150 mm at the ages of 7-day and 28-day according to ECP 203^24^, the results are shown in Table 4.

**Fig. 6:**
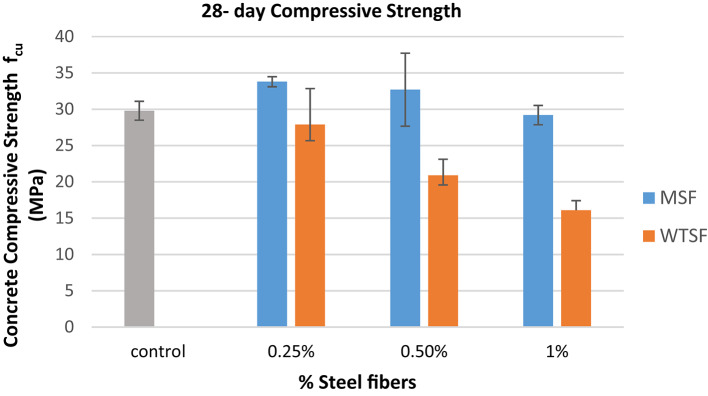
compressive strength of different concrete mixtures at the age of 28-day.

**Fig. 7:**
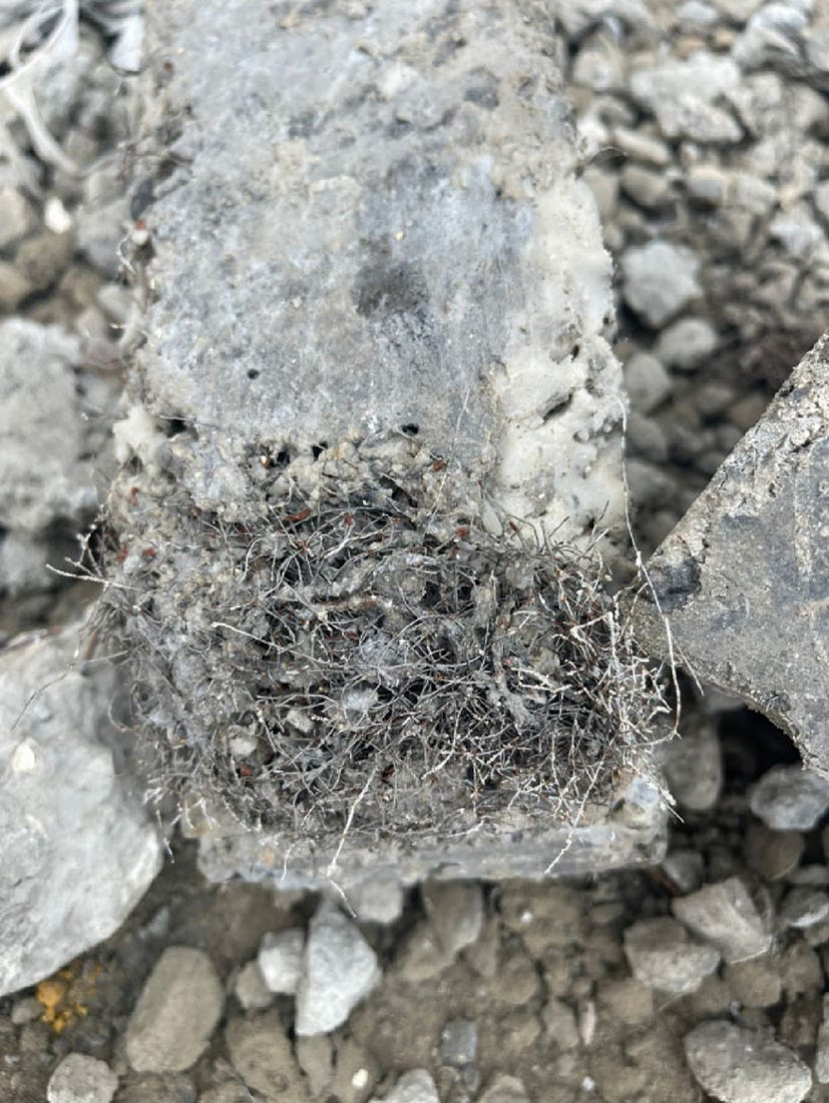
Clumps formed of Waste Tires Steel Fibers (WTSF).

**Fig. 8:**
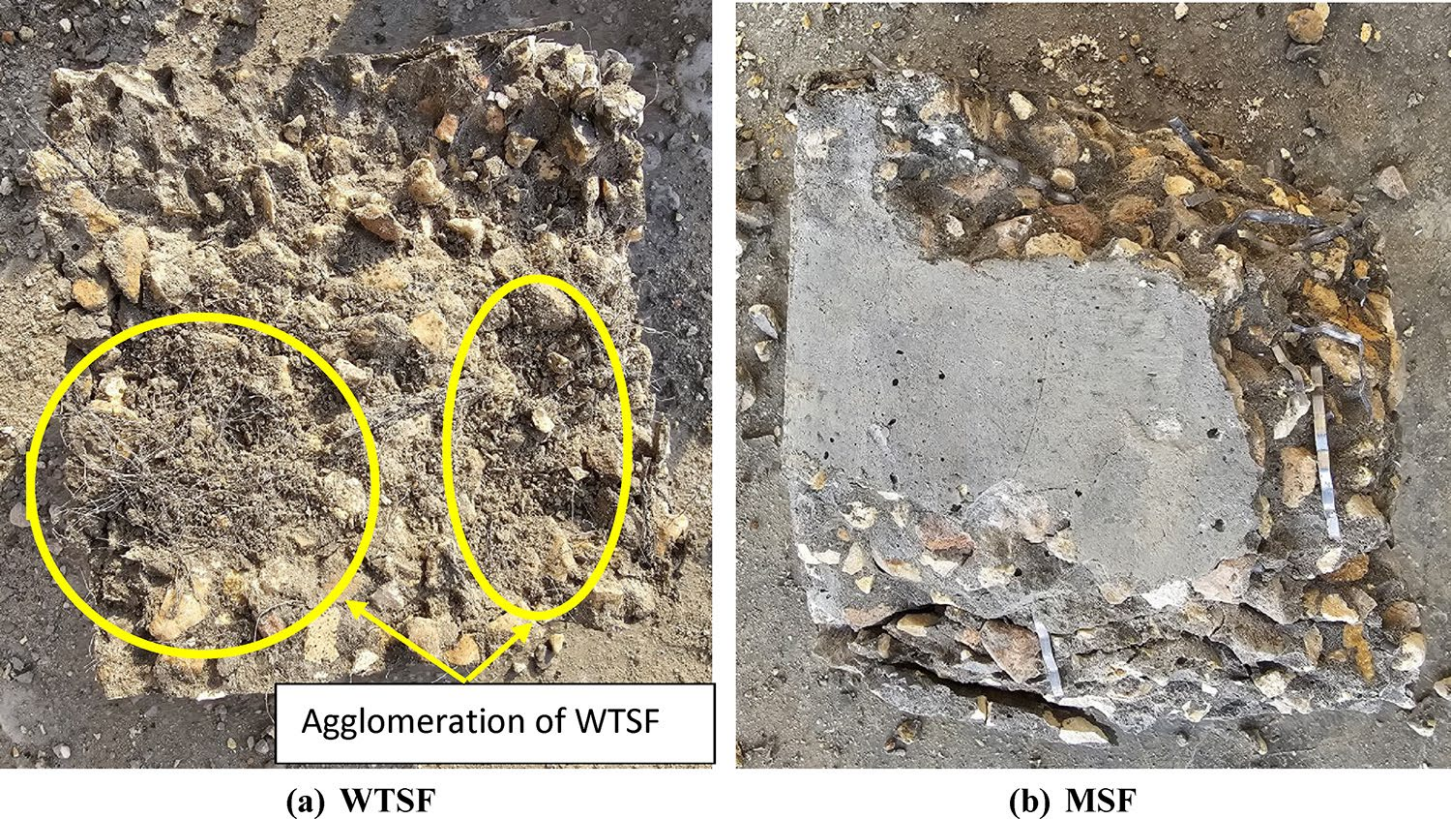
Failure modes of concrete reinforced with steel fibers.

The results showed that the use of MSF slightly improved the compressive strength of the concrete mix at both ages (7 and 28 days), but on the other hand, the use of WTSF reduced the compressive strength of the concrete significantly, especially at higher percentages. Figure 6 illustrates the compressive strength of different concrete mixtures at the age of 28-day. When MSF was incorporated into the concrete mix at volume ratios of 0.25% and 0.5%, the compressive strength of the concrete increased by 13.5% and 9.8%, respectively, in comparison to the control sample at the age of 28 days. However, increasing the MSF content to 1% resulted in no significant change in compressive strength relative to the control sample at 28 days. In contrast, the addition of WTSF at volume ratios of 0.25%, 0.5%, and 1% led to reductions in compressive strength of 6.4%, 30%, and 46%, respectively, compared to the control sample at the same age of 28 days. The reduction in compressive strength of mixtures containing WTSF can be attributed to several factors. Due to the very high aspect ratio of the waste steel fiber, it tends to form clumps within the mix. These clumps prevent uniform fiber distribution and create localized spots of honeycombs, which can initiate cracks when the concrete is subjected to compressive loads. As a result of increasing the percentages of WTSF in the concrete mix, the possibility of having such clumps increases. This leads to a further decrease in compressive strength, as observed in the test results. Figure 7 shows the Clumps formed by waste tires steel fibers (WTSF). Another reason for such a drop in compressive strength is the residual rubber that sticks to the steel fibers during its extraction in the recycling process; such rubber residue has a weak bond with the concrete materials, which negatively affects the compressive strength. It can be observed that increasing the WTSF content from 0.25 to 1% led to a greater formation of fiber clumps, which negatively affected the compressive strength. As a result, the compressive strength decreased from 6.4 to 46% compared to the control mix. In other words, increasing the fiber content by 0.75% resulted in an overall reduction of compressive strength by approximately 39.6%. These findings are consistent with those reported by Papakonstantinou et al.^21^ and Leone et al.^22^ who also observed that the addition of WTSF can lead to further reductions in compressive strength. Additionally, Zia et al.^18^ noted that the rate of compressive strength reduction increases as the ratio of WTSF in the concrete mix increases. Therefore, this study contributes to resolving the contradictory findings in the literature by providing further evidence that WTSF can adversely affect the compressive strength of concrete, especially at higher ratios. Figure 8 shows the difference between the failure modes of 1% WTSF and 1% MSF.

**Table 4:** Compressive strength of specimens.

Mix name	7-day Compressive strength (MPa)	28-day Compressive strength (MPa)
Control	20.9$\:\pm\:$ 1.4	29.79$\:\pm\:$1.3
MSF (0.25%)	27.09$\:\pm\:$ 0.31	33.79$\:\pm\:$0.69
MSF (0.50%)	22.89$\:\pm\:$0.50	32.69$\:\pm\:$5.03
MSF (1.0%)	22.59$\:\pm\:$1.34	29.19$\:\pm\:$1.33
WTSF (0.25%)	18.49$\:\pm\:$0.91	27.89$\:\pm\:4.95$
WTSF (0.50%)	15.49$\:\pm\:$1.4	20.89$\:\pm\:$2.22
WTSF (1.0%)	12.79$\:\pm\:$0.1	16.09$\:\pm\:$1.32

### Tensile strength

The indirect splitting tensile strength test was carried out on standard cylinders (150 × 300 mm) at 28-day age, the test was carried out in accordance with ASTM C496/C496M-11^28^. Table 5 shows the results of the tensile strength test of the concrete mixtures.

**Fig. 9:**
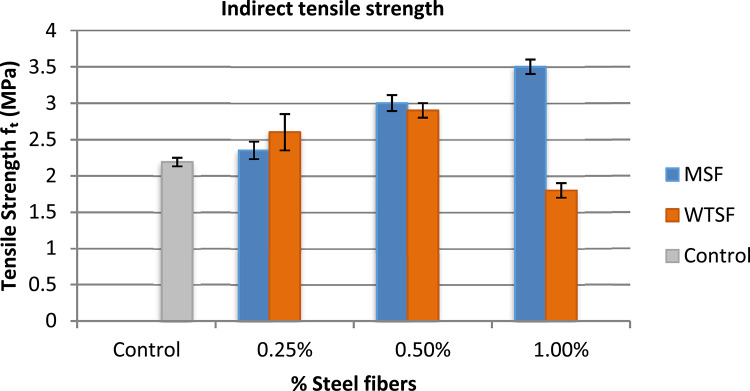
Indirect tensile strength of different concrete mixtures.

The results showed a significant increase in the tensile strength of concrete when the steel fibers were added to the concrete mix. Figure 9 illustrates the indirect tensile strength of different concrete mixtures compared to the control mix. For the MSF, the increase in tensile strength compared to the control mix ranged from 10 to 66.7% for concrete mixtures with MSF ratios of 0.25% and 1%, respectively. In contrast, the results for WTSF displayed greater variability, with tensile strength increases of 24% and 38% for WTSF ratios of 0.25% and 0.5%, respectively, compared to the control mix. However, when the WTSF content was increased to 1% by volume, the tensile strength decreased by 10%.

**Fig. 10:**
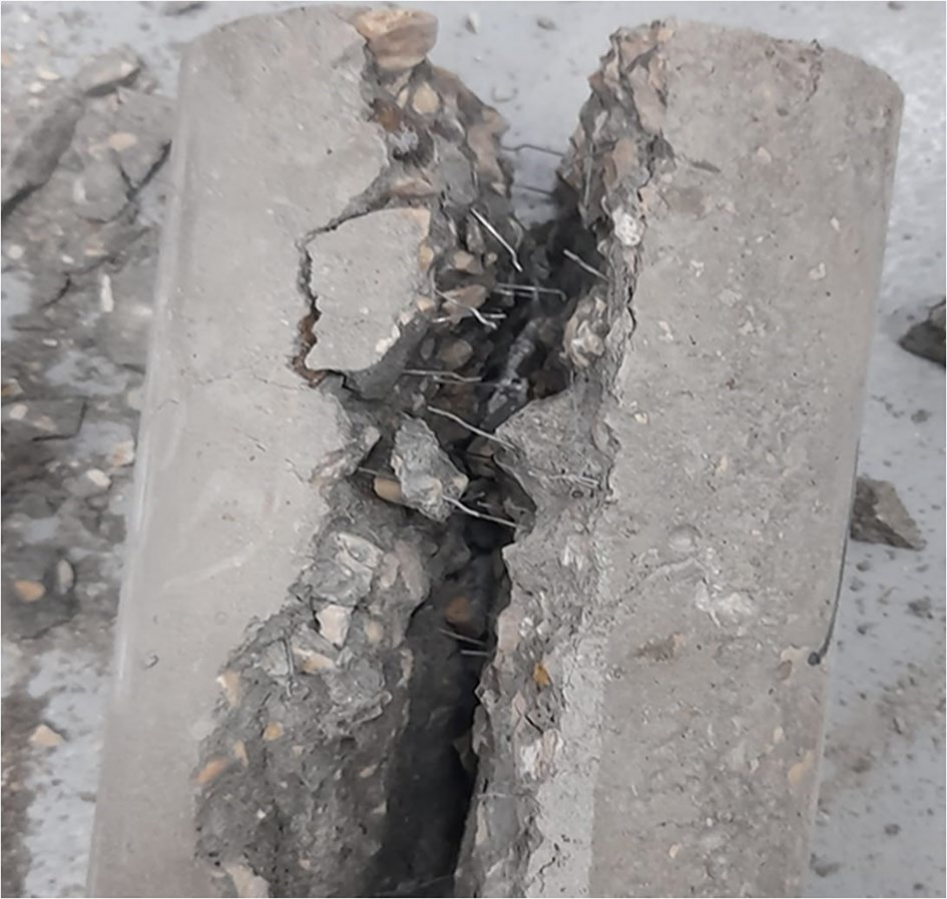
Splitting tensile failure mode for concrete with WTSF.

This can be explained as by the addition of steel fibers to plain concrete, where the fibers were able to withstand and carry the tensile stresses developed in the concrete, thereby delaying the initiation of tensile cracks. As the percentage of steel fibers increases, the tensile strength of the concrete improves accordingly. However, in the case of the 1% WTSF, the high fiber content in the concrete mix prevented uniform distribution, resulting in the formation of fiber agglomeration in various locations of the samples, which led to a reduction in strength. Figure 10 Shows the failure mode of the splitting tensile sample. Similar results were reported by Zia et al.^18^, who found that as the fiber content increased, the tensile splitting strengths also improved.

**Table 5:** Tensile strength of different concrete mixtures.

Mix	Indirect tensile strength (MPa)
Control	2.19$\:\pm\:$0.06
MSF (0.25%)	2.39$\:\pm\:$0.0
MSF (0.50%)	3.09$\:\pm\:$0.12
MSF (1.0%)	3.5$\:\pm\:$0.25
WTSF (0.25%)	2.6$\:\pm\:$0.11
WTSF (0.50%)	2.9$\:\pm\:$0.10
WTSF (1.0%)	1.8$\:\pm\:$0.10

### Flexural strength

Flexural tests were carried out on the specimens after 28-day age, the test was carried out in accordance with ASTM C78/C78M-18^29^. Table 6 shows the results of the flexural strength of different concrete mixtures.

**Fig. 11:**
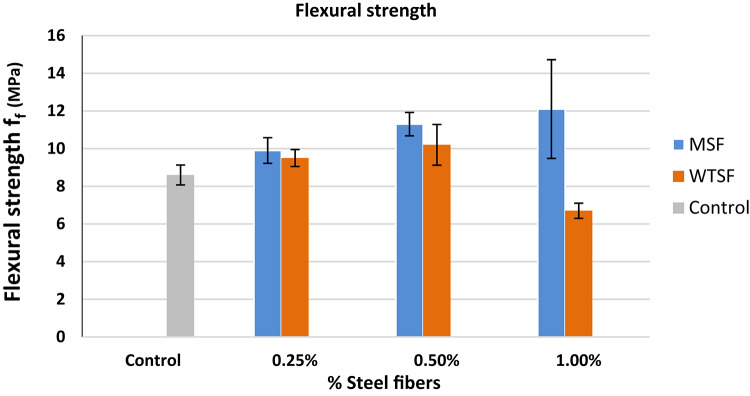
Flexural strength of different concrete mixtures.

Adding the steel fibers to the concrete mixture showed a significant increase in the flexural strength of concrete as the steel fibers were able to carry the tensile forces developed at the bottom of the beam specimens and enhance the post-cracking behaviour of the specimens. Figure 11 illustrates the flexural strength of different concrete mixtures compared to the control mix The MSF showed a superior behaviour compared to that of the control specimens and the WTSF specimens. The addition of WTSF at ratios of 0.25% and 0.5% resulted in flexural strength increases of 10% and 19%, respectively, similar results were obtained by Ali Elrefai et al.^30^. The maximum increase in flexural strength, 40%, was achieved with the MSF content ratio of 1%.

Alike the behavior in compression and splitting tension, the specimens of 1% WTSF showed a sudden drop in the strength due to the formation of internal weak spots in the specimens created from the clumps of the WTSF.

**Table 6:** Flexural strength of different concrete mixtures.

Mix	Flexural strength (MPa)
Control	8.6$\:\pm\:$0.53
MSF (0.25%)	9.9$\:\pm\:$0.68
MSF (0.50%)	11.3$\:\pm\:$0.62
MSF (1.0%)	12.1$\:\pm\:$2.62
WTSF (0.25%)	9.5$\:\pm\:$0.45
WTSF (0.50%)	10.2$\:\pm\:$1.08
WTSF (1.0%)	6.7$\:\pm\:$0.40

## Conclusions

This study investigated the effect of adding different forms of steel wires on the properties of fresh and hardened concrete, where two different types of steel fibers were used: manufactured steel fibers (MSF) and Waste Tire Steel Fibers (WTSF).

The steel fibers were used as a percentage of the concrete by volume where the used percentages were 0.25%, 0.5% and 1%, based on the results obtained from this study the following conclusions can be drawn:

- The use of different types of steel fibers reduces the workability of concrete significantly, where WTSF has severer effect than MSF.
- There is a slight variation in compressive strength when MSF is used in the concrete mixes. In mixes with low content of MSF ratios of 0.25% and 0.5%, the compressive strength increases slightly by 13.5% and 9.8%, respectively, in comparison to the control sample at the age of 28 days. However, increasing the MSF content to 1% resulted in a slight degradation in the compressive strength. In conclusion, the variation in compressive strength was insignificant for all cases.
- In contrast, using WTSF negatively affected the compressive strength of concrete especially at higher ratios of WTSF content. The addition of WTSF at volume ratios of 0.25%, 0.5%, and 1% led to reductions in compressive strength of 6.4%, 30%, and 46%, respectively, compared to the control sample at the same age of 28 days.
- The use of MSF and WTSF enhanced the tensile strength of concrete significantly, where a 67% increase was achieved when the MSF ratio reached 1%, while a 38% increase was achieved when the WTSF ratio reached 0.50%.
- The flexural strength was improved by 40% when the MSF ratio reached 1%, while for the mixes with WTSF, the maximum improvement was 19%, corresponding to 0.5% of WTSF content.
- The results showed that the MSF had superior behaviour compared to that of WTSF, but when considering the economic and environmental aspects, the use of WTSFcan be a satisfactory choice for enhancing the tensile and flexural behaviour of concrete.

Due to the observed reduction in compressive strength and the potential for fiber agglomeration at higher contents, the use of WTSF should be limited to non-load-bearing or secondary structural components, such as pavements, partitions, or non-structural walls. These findings suggest that WTSF is not suitable for primary load-bearing applications. Further studies are recommended to optimize fiber content and enhance the uniformity of fiber distribution to mitigate agglomeration and improve the mechanical properties of the resulting concrete.

## Data Availability

All data generated or analyzed during this study are included in this published article.
